# Supplementary material for: Generating New FANCA-Deficient HNSCC Cell Lines by Genomic Editing Recapitulates the Cellular Phenotypes of Fanconi Anemia
Source: Genes (Basel). 2021 Apr 9;12(4):548. doi: 10.3390/genes12040548 (PMC8069753; doi:10.3390/genes12040548)
Supplement: Supplementary file 1 [file genes-12-00548-s001.pdf]

FIGURE S1

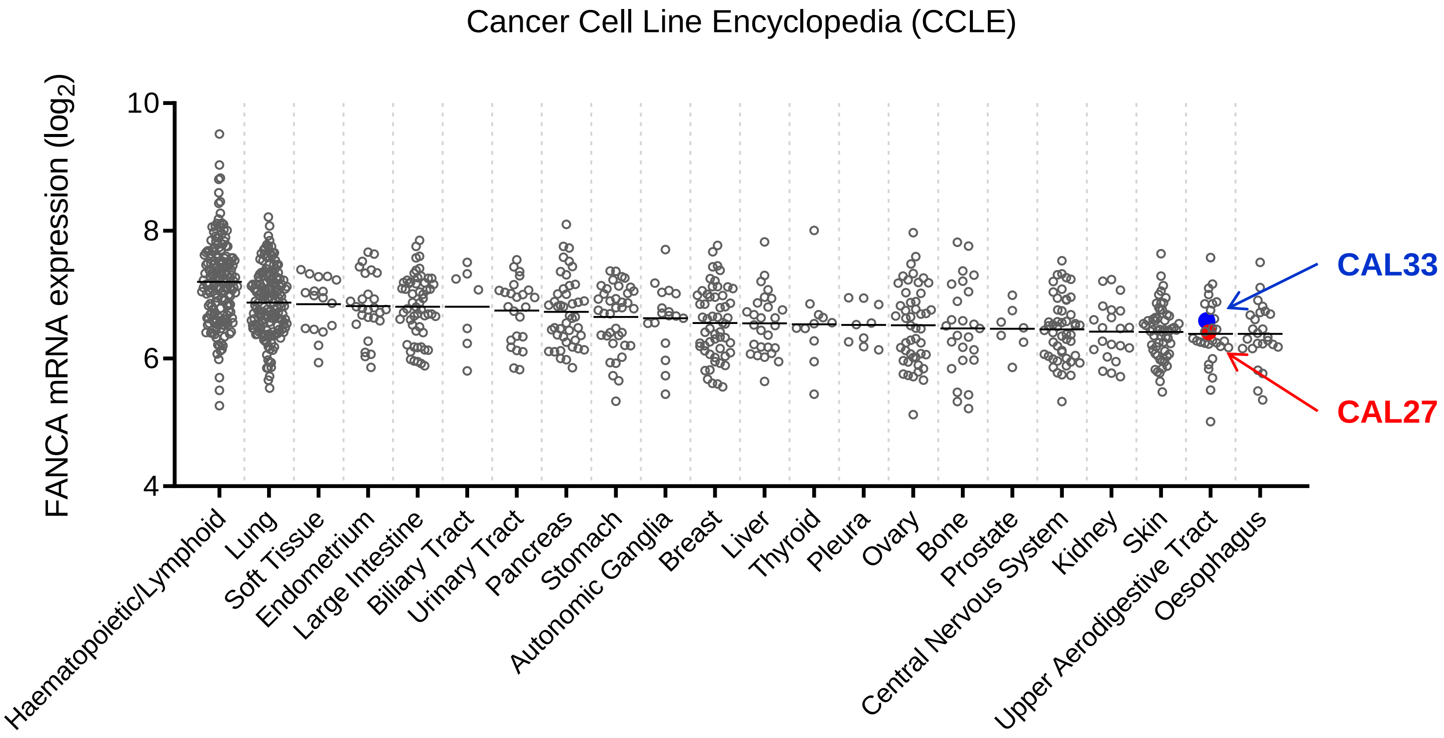

Figure S1. Expression values of FANCA gene mRNA from The Cancer Cell Line Encyclopedia collection. Cell lines are grouped by tissue location. Expression value positions of CAL27 and CAL33 cells are shown.

FIGURE S2

A Sequence position of gGM10 guide RNA within FANCA exon4

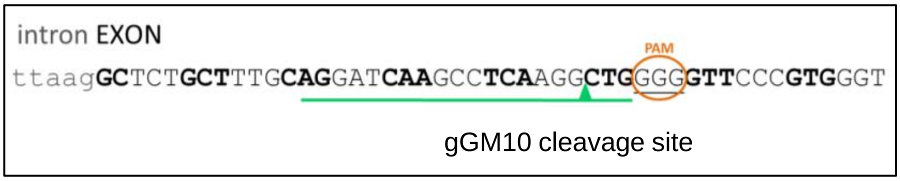

B Editing of CAL27-c34 clone: 2 nucleotide deletion in FANCA ORF

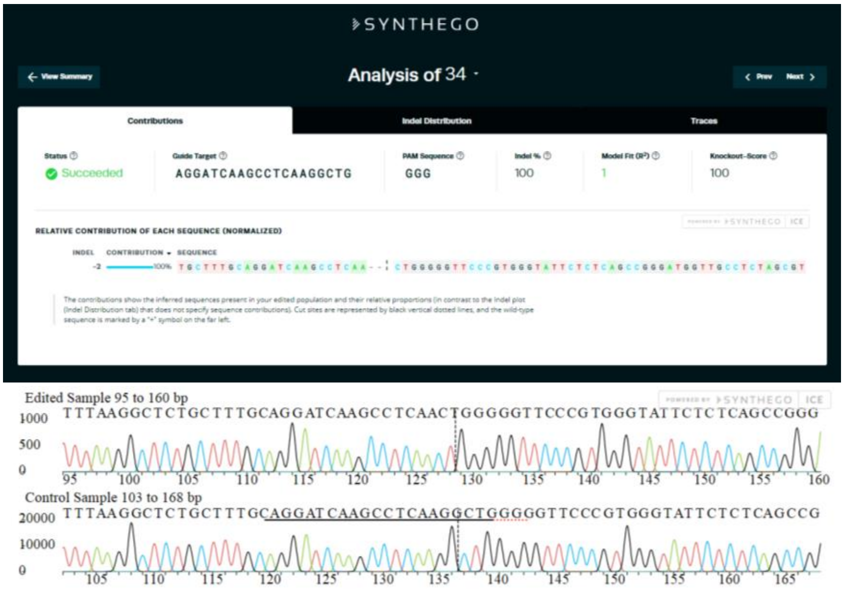

C Editing of CAL27-c47 clone: 14 nucleotide deletion in FANCA ORF

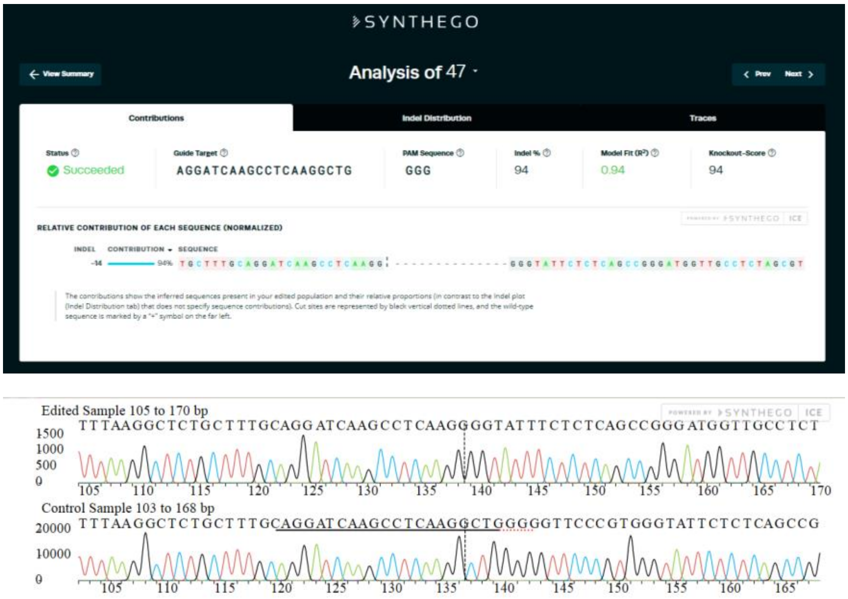

Figure S2. A) Recognition sequence and cleavage site of guide RNA gGM10 (green) in the exon 4 of the FANCA gene, 3 nucleotides before the PAM-sequence (orange). **B)** Editing analysis of clon 34 from CAL27 (CAL27-c34) after Sanger sequencing. **C)** Editing analysis of clon 47 from CAL27 (CAL27-c47) after Sanger sequencing.

FIGURE S3

A Editing of CAL33-c5 clone: 2 nucleotide deletion in FANCA ORF

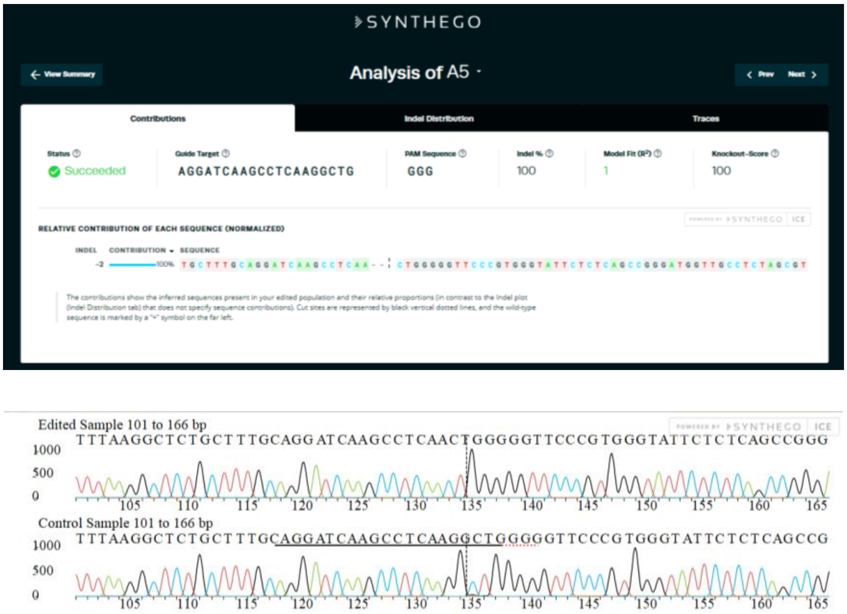

B Editing of CAL33-c11 clone: 17 nucleotide deletion in FANCA ORF

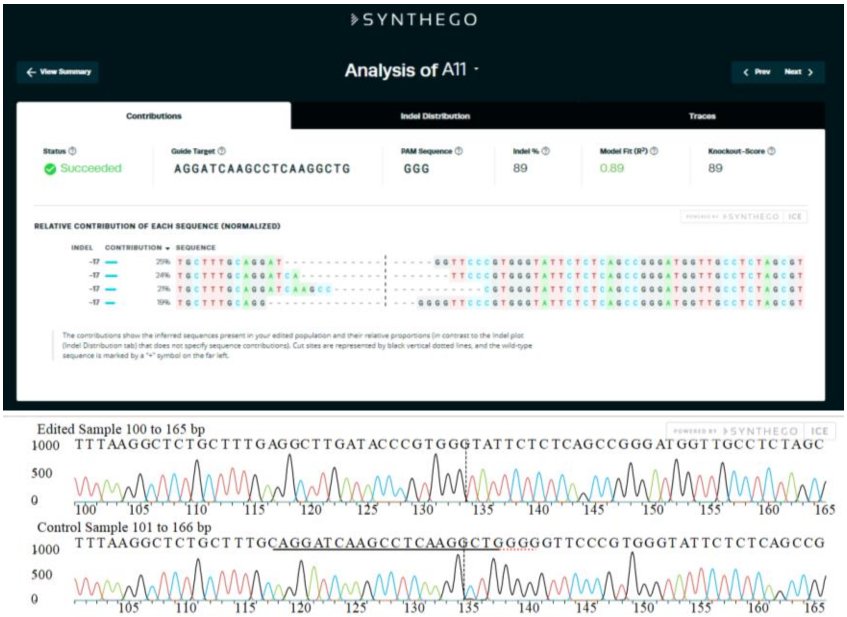

Figure S3. A) Editing analysis of clon 5 from CAL33 (CAL33-c5) after Sanger sequencing. **B)** Editing analysis of clon 11 from CAL33 (CAL33-c11) after Sanger sequencing.

FIGURE S4

A Non-editing of CAL27-c27 clone: wt sequence in FANCA ORF

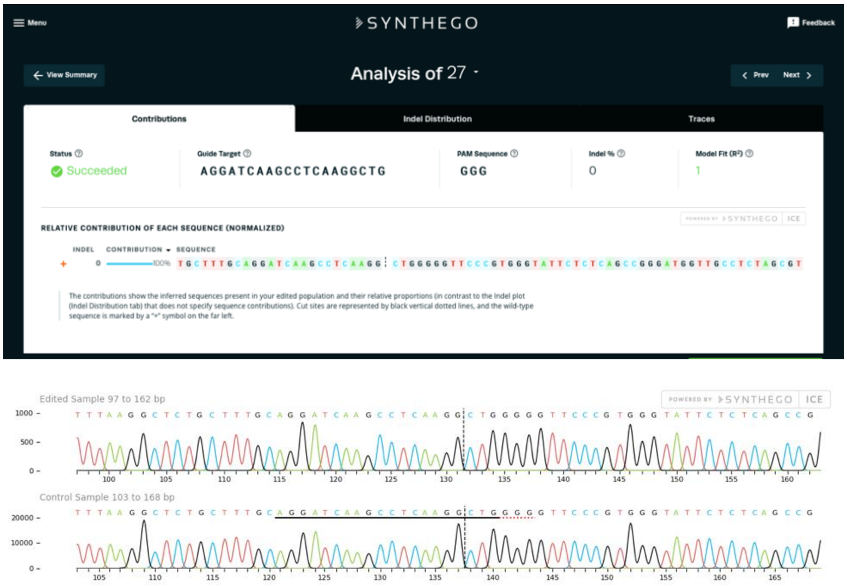

B Non-editing of CAL33-c18 clone: wt sequence in FANCA ORF

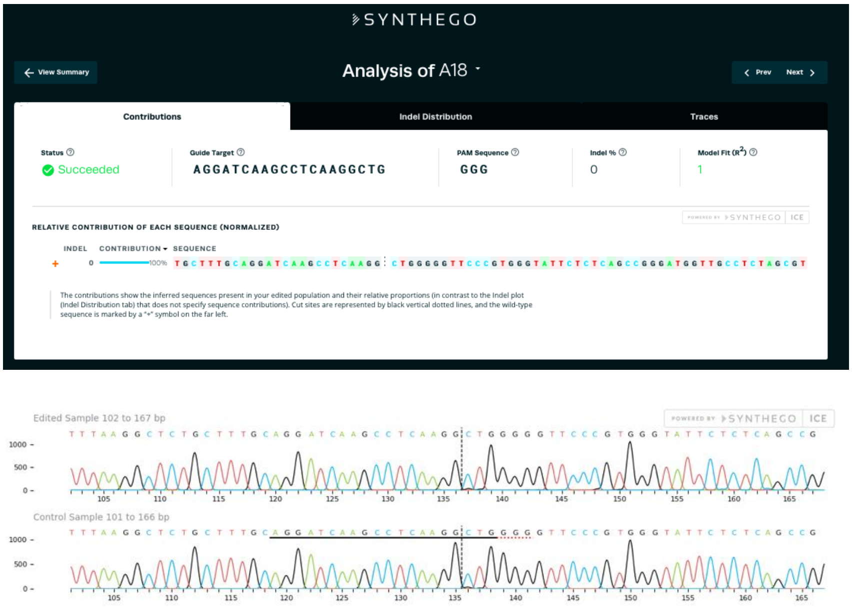

Figure S4. A) Non-editing analysis of clon 27 from CAL27 (CAL27-c27) after Sanger sequencing. **B)** Non-editing analysis of clon 18 from CAL33 (CAL33-c18) after Sanger sequencing.

FIGURE S5

A

MMC sensitivity profiles of CAL27 parental, non-edited and edited clones, and FANCA-complemented clones

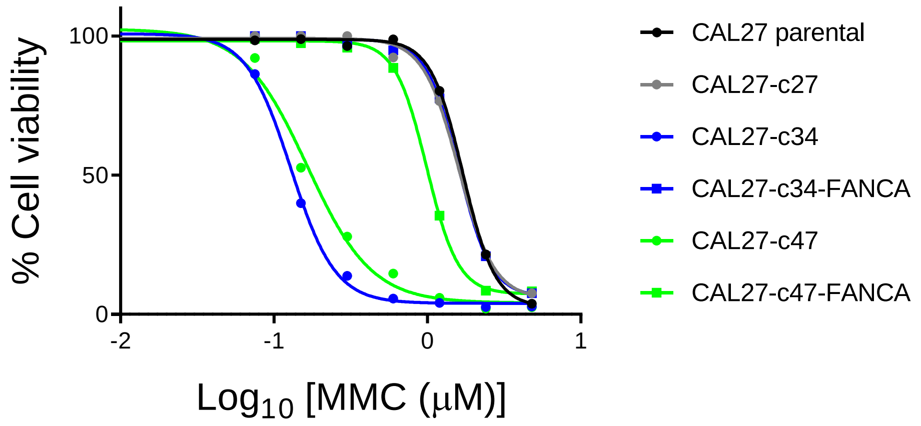

B

MMC sensitivity profiles of CAL33 parental, non-edited and edited clones, and FANCA-complemented clones

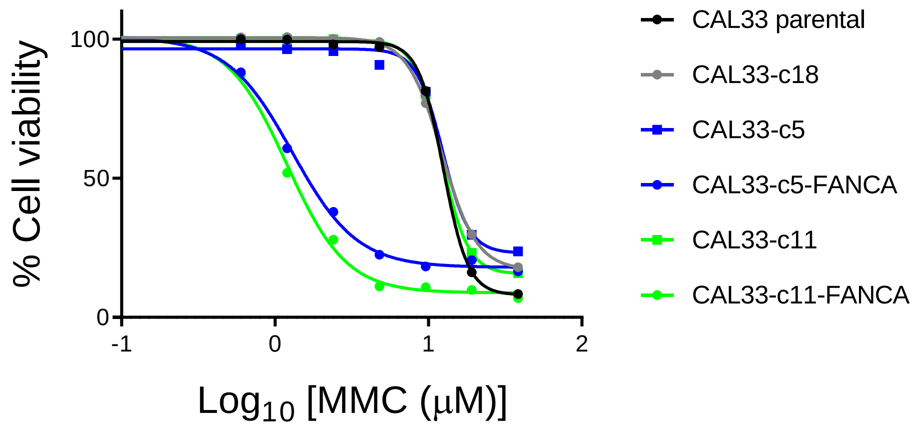

Figure S5. Non-edited CAL27-c27 **(A)** and CAL33-c18 **(B)** clones are resistant to MMC treatment as their parental counterparts. FANCA-edited mutant CAL27 **(A)** and CAL33 **(B)** clones were complemented with wild type FANCA gene and treated with MMC. Complemented cells displayed similar IC50 values than parental cells. Cells were treated with increasing concentrations of MMC for 1 hour and grown until control cells reached the maximum confluence (5 days). Cells were stained with crystal violet, eluted with acetic acid, and color intensity was measured with absorbance at 620 nm. Data represent means ± SEMs from three different experiments for each cell line.

Table S1. Gene expression values and copy-number status of the FANCA gene in the Cancer Cell Line Enciclopedia (CCLE) collection.

Sample Id	FANCA, Putative copy-number alterations	FANCA, mRNA expression (RNA-Seq RPKM)
A101D_SKIN	0	6.71143
A1207_CENTRAL_NERVOUS_SYSTEM	0	10.4597
A172_CENTRAL_NERVOUS_SYSTEM	0	9.54414
A204_SOFT_TISSUE	0	8.5877
A2058_SKIN	0	15.31827
A253_SALIVARY_GLAND	0	2.2111
A2780_OVARY	0	13.14268
A375_SKIN	0	9.22497
A3KAW_HAEMATOPOIETIC_AND_LYMPHOID_TISSUE	0	3.52577
A498_KIDNEY	0	5.31945
A4FUK_HAEMATOPOIETIC_AND_LYMPHOID_TISSUE	0	12.98519
A549_LUNG	0	7.10322
A673_BONE	0	16.16693
A704_KIDNEY	0	5.84158
ABC1_LUNG	0	7.30138
ACCMESO1_PLEURA	0	5.1411
ACHN_KIDNEY	0	13.23184
AGS_STOMACH	0	12.61685
ALLSIL_HAEMATOPOIETIC_AND_LYMPHOID_TISSUE	0	18.88077
AM38_CENTRAL_NERVOUS_SYSTEM	0	13.02183
AML193_HAEMATOPOIETIC_AND_LYMPHOID_TISSUE	0	18.44638
AMO1_HAEMATOPOIETIC_AND_LYMPHOID_TISSUE	0	11.11269
AN3CA_ENDOMETRIUM	0	18.40104
ASPC1_PANCREAS	0	2.5253
AU565_BREAST	-2	5.90435
BC3C_URINARY_TRACT	0	13.0205
BCP1_HAEMATOPOIETIC_AND_LYMPHOID_TISSUE	0	13.32998
BCPAP_THYROID	0	1.27261
BDCM_HAEMATOPOIETIC_AND_LYMPHOID_TISSUE	0	15.99499
BECKER_CENTRAL_NERVOUS_SYSTEM	0	NA
BEN_LUNG	0	12.38663
BFTC905_URINARY_TRACT	0	10.15519
BFTC909_KIDNEY	0	7.99318
BHT101_THYROID	0	8.32884
BHY_UPPER_AERODIGESTIVE_TRACT	-2	4.80829
BICR16_UPPER_AERODIGESTIVE_TRACT	2	7.07537
BICR18_UPPER_AERODIGESTIVE_TRACT	0	8.02305
BICR22_UPPER_AERODIGESTIVE_TRACT	0	1.14639
BICR31_UPPER_AERODIGESTIVE_TRACT	0	5.20502
BICR56_UPPER_AERODIGESTIVE_TRACT	0	0.39535
BICR6_UPPER_AERODIGESTIVE_TRACT	0	4.06894
BL41_HAEMATOPOIETIC_AND_LYMPHOID_TISSUE	0	32.78154
BL70_HAEMATOPOIETIC_AND_LYMPHOID_TISSUE	0	23.39094
BT20_BREAST	2	18.66049
BT474_BREAST	0	7.90303
BT483_BREAST	0	4.92945
BT549_BREAST	0	10.05888
BV173_HAEMATOPOIETIC_AND_LYMPHOID_TISSUE	0	17.34491
BXPC3_PANCREAS	0	8.91589
C2BBE1_LARGE_INTESTINE	0	15.65722
C32_SKIN	0	10.44541
C3A_LIVER	0	NA
CA46_HAEMATOPOIETIC_AND_LYMPHOID_TISSUE	0	49.64153
CADOES1_BONE	0	8.15332
CAKI1_KIDNEY	0	3.69058
CAKI2_KIDNEY	0	10.14309
CAL120_BREAST	0	3.75539
CAL12T_LUNG	0	9.10623
CAL148_BREAST	-2	5.1111
CAL27_UPPER_AERODIGESTIVE_TRACT	0	5.91309
CAL29_URINARY_TRACT	0	10.22204
CAL33_UPPER_AERODIGESTIVE_TRACT	0	6.55359
CAL51_BREAST	0	25.84445
CAL54_KIDNEY	0	7.30838
CAL62_THYROID	0	21.54635
CAL78_BONE	-2	5.56705
CAL851_BREAST	0	4.75151
CALU1_LUNG	0	9.32507
CALU3_LUNG	0	9.93475
CALU6_LUNG	0	12.6772
CAMA1_BREAST	0	11.13695
CAOV3_OVARY	0	3.92463
CAOV4_OVARY	0	5.41441
CAPAN1_PANCREAS	2	20.41831
CAPAN2_PANCREAS	0	15.79056
CAS1_CENTRAL_NERVOUS_SYSTEM	0	4.63457

CCFSTTG1_CENTRAL_NERVOUS_SYSTEM	0	15.18541
CCK81_LARGE_INTESTINE	0	23.03826
CFPAC1_PANCREAS	0	4.68861
CGTHW1_THYROID	0	NA
CH157MN_CENTRAL_NERVOUS_SYSTEM	0	7.27462
CHAGOK1_LUNG	0	21.93406
CHL1_SKIN	0	NA
CHP126_AUTONOMIC_GANGLIA	0	10.33352
CHP212_AUTONOMIC_GANGLIA	-2	3.34798
CI1_HAEMATOPOIETIC_AND_LYMPHOID_TISSUE	0	21.60964
CJM_SKIN	0	3.72168
CL11_LARGE_INTESTINE	0	7.25958
CL14_LARGE_INTESTINE	0	4.83751
CL34_LARGE_INTESTINE	0	14.18198
CL40_LARGE_INTESTINE	0	3.80581
CMK_HAEMATOPOIETIC_AND_LYMPHOID_TISSUE	0	10.19759
CMK115_HAEMATOPOIETIC_AND_LYMPHOID_TISSUE	0	NA
CMLT1_HAEMATOPOIETIC_AND_LYMPHOID_TISSUE	0	8.9651
COLO205_LARGE_INTESTINE	0	NA
COLO320_LARGE_INTESTINE	0	15.43416
COLO668_LUNG	0	26.72075
COLO678_LARGE_INTESTINE	0	8.03877
COLO679_SKIN	0	9.48422
COLO680N_OESOPHAGUS	0	3.66032
COLO684_ENDOMETRIUM	0	19.4182
COLO704_OVARY	0	NA
COLO741_SKIN	0	8.65234
COLO775_HAEMATOPOIETIC_AND_LYMPHOID_TISSUE	0	NA
COLO783_SKIN	0	5.31422
COLO792_SKIN	-2	3.96528
COLO800_SKIN	0	8.0196
COLO818_SKIN	0	NA
COLO829_SKIN	0	2.4749
CORL105_LUNG	0	8.80936
CORL23_LUNG	0	6.58461
CORL24_LUNG	-2	12.96881
CORL279_LUNG	0	11.02129
CORL311_LUNG	0	11.2869
CORL47_LUNG	0	23.68171
CORL51_LUNG	0	NA
CORL88_LUNG	0	27.20511
CORL95_LUNG	0	11.70101
COV318_OVARY	0	6.58902
COV362_OVARY	0	3.49105
COV434_OVARY	0	12.78215
COV504_OVARY	0	5.7998
COV644_OVARY	0	7.92203
CPCN_LUNG	0	NA
CW2_LARGE_INTESTINE	0	11.59826
D283MED_CENTRAL_NERVOUS_SYSTEM	-2	10.92657
D341MED_CENTRAL_NERVOUS_SYSTEM	0	5.88443
DANG_PANCREAS	0	10.78548
DAOY_CENTRAL_NERVOUS_SYSTEM	0	10.70883
DAUDI_HAEMATOPOIETIC_AND_LYMPHOID_TISSUE	0	80.70203
DB_HAEMATOPOIETIC_AND_LYMPHOID_TISSUE	0	17.18457
DBTRG05MG_CENTRAL_NERVOUS_SYSTEM	0	3.70703
DEL_HAEMATOPOIETIC_AND_LYMPHOID_TISSUE	0	7.33995
DETROIT562_UPPER_AERODIGESTIVE_TRACT	0	10.10715
DKMG_CENTRAL_NERVOUS_SYSTEM	0	6.37161
DMS114_LUNG	0	14.08016
DMS153_LUNG	0	18.15383
DMS273_LUNG	0	17.88724
DMS454_LUNG	0	18.95676
DMS53_LUNG	0	10.51834
DMS79_LUNG	0	14.6853
DND41_HAEMATOPOIETIC_AND_LYMPHOID_TISSUE	0	13.57075
DOHH2_HAEMATOPOIETIC_AND_LYMPHOID_TISSUE	0	40.68032
DOV13_OVARY	0	NA
DU145_PROSTATE	0	12.71358
DU4475_BREAST	0	9.47227
DV90_LUNG	0	17.56098
EB1_HAEMATOPOIETIC_AND_LYMPHOID_TISSUE	0	10.85989
EB2_HAEMATOPOIETIC_AND_LYMPHOID_TISSUE	0	NA
EBC1_LUNG	0	14.83171
ECC10_STOMACH	-2	7.9406
ECC12_STOMACH	0	15.46928
ECGI10_OESOPHAGUS	0	3.76488
EFE184_ENDOMETRIUM	2	6.56725
EFM19_BREAST	0	4.43397

EFM192A_BREAST	0	10.95299
EFO21_OVARY	0	4.80802
EFO27_OVARY	0	7.74949
EHEB_HAEMATOPOIETIC_AND_LYMPHOID_TISSUE	0	6.31866
EJM_HAEMATOPOIETIC_AND_LYMPHOID_TISSUE	0	7.967
EKVX_LUNG	0	10.32401
EM2_HAEMATOPOIETIC_AND_LYMPHOID_TISSUE	-2	9.36041
EN_ENDOMETRIUM	0	8.17147
EOL1_HAEMATOPOIETIC_AND_LYMPHOID_TISSUE	0	11.78547
EPLC272H_LUNG	0	9.2837
ES2_OVARY	0	9.02234
ESS1_ENDOMETRIUM	0	13.34832
EVSAT_BREAST	0	NA
EW8_BONE	0	14.84115
EWS502_BONE	0	21.19112
F36P_HAEMATOPOIETIC_AND_LYMPHOID_TISSUE	0	7.43034
FADU_UPPER_AERODIGESTIVE_TRACT	0	6.09374
FTC133_THYROID	0	4.97391
FTC238_THYROID	0	7.21639
FU97_STOMACH	0	11.19052
FUOV1_OVARY	0	10.45671
G292CLONEA141B1_BONE	0	7.39025
G361_SKIN	0	6.92436
G401_SOFT_TISSUE	0	12.61644
G402_SOFT_TISSUE	0	9.87264
GA10_HAEMATOPOIETIC_AND_LYMPHOID_TISSUE	2	55.58822
GAMG_CENTRAL_NERVOUS_SYSTEM	0	9.52241
GB1_CENTRAL_NERVOUS_SYSTEM	0	6.54245
GCIY_STOMACH	0	8.5518
GCT_SOFT_TISSUE	0	13.80771
GDM1_HAEMATOPOIETIC_AND_LYMPHOID_TISSUE	0	11.59315
GI1_CENTRAL_NERVOUS_SYSTEM	0	8.97529
GMS10_CENTRAL_NERVOUS_SYSTEM	0	11.40363
GOS3_CENTRAL_NERVOUS_SYSTEM	0	5.31892
GP2D_LARGE_INTESTINE	0	24.34428
GRANTA519_HAEMATOPOIETIC_AND_LYMPHOID_TISSUE	0	16.18714
GRM_SKIN	0	NA
GSS_STOMACH	0	11.4566
GSU_STOMACH	0	4.21369
H4_CENTRAL_NERVOUS_SYSTEM	0	11.9234
HARA_LUNG	0	14.89824
HCC1143_BREAST	0	15.03464
HCC1171_LUNG	0	8.21191
HCC1187_BREAST	0	8.15366
HCC1195_LUNG	-2	3.5637
HCC1359_LUNG	0	10.62774
HCC1395_BREAST	0	10.75934
HCC1419_BREAST	-2	4.83442
HCC1428_BREAST	0	5.52218
HCC15_LUNG	0	21.97591
HCC1500_BREAST	0	8.6314
HCC1569_BREAST	0	7.64915
HCC1599_BREAST	0	14.0451
HCC1806_BREAST	0	5.1049
HCC1937_BREAST	0	14.12827
HCC1954_BREAST	0	13.75857
HCC202_BREAST	0	4.53273
HCC2157_BREAST	0	7.60762
HCC2218_BREAST	-2	1.95714
HCC2279_LUNG	0	4.94245
HCC2814_LUNG	0	15.19688
HCC2935_LUNG	0	9.7464
HCC33_LUNG	0	20.80921
HCC364_LUNG	-2	5.06191
HCC366_LUNG	0	10.32446
HCC38_BREAST	0	8.79967
HCC4006_LUNG	0	8.15579
HCC44_LUNG	0	8.35347
HCC56_LARGE_INTESTINE	0	8.63477
HCC70_BREAST	0	13.70732
HCC78_LUNG	0	12.02369
HCC827_LUNG	0	9.0649
HCC95_LUNG	0	9.46509
HCT116_LARGE_INTESTINE	0	23.06592
HCT15_LARGE_INTESTINE	0	13.68639
HDLM2_HAEMATOPOIETIC_AND_LYMPHOID_TISSUE	-2	6.65299
HDMYZ_HAEMATOPOIETIC_AND_LYMPHOID_TISSUE	0	8.77055
HDQP1_BREAST	0	3.9161
HEC108_ENDOMETRIUM	0	19.01297

HEC151_ENDOMETRIUM	0	18.86024
HEC1A_ENDOMETRIUM	0	10.50695
HEC1B_ENDOMETRIUM	0	10.20616
HEC251_ENDOMETRIUM	0	8.96978
HEC265_ENDOMETRIUM	0	21.48343
HEC50B_ENDOMETRIUM	0	9.03605
HEC59_ENDOMETRIUM	0	13.14463
HEC6_ENDOMETRIUM	0	13.38916
HEKTE_KIDNEY	0	10.30977
HEL_HAEMATOPOIETIC_AND_LYMPHOID_TISSUE	0	5.93573
HEL9217_HAEMATOPOIETIC_AND_LYMPHOID_TISSUE	0	7.42995
HEP3B217_LIVER	0	9.93875
HEPG2_LIVER	0	13.79642
HEYA8_OVARY	0	4.20202
HGC27_STOMACH	0	11.12823
HH_HAEMATOPOIETIC_AND_LYMPHOID_TISSUE	0	12.77065
HL60_HAEMATOPOIETIC_AND_LYMPHOID_TISSUE	-2	7.63149
HLE_LIVER	0	NA
HLF_LIVER	0	11.16479
HMC18_BREAST	0	11.13475
HMCB_SKIN	0	9.59459
HMEL_BREAST	0	6.38055
HN_UPPER_AERODIGESTIVE_TRACT	0	NA
HOP62_LUNG	0	16.869
HOP92_LUNG	0	4.16854
HOS_BONE	0	11.67302
HPAC_PANCREAS	0	9.35961
HPAFII_PANCREAS	0	5.67978
HPBALL_HAEMATOPOIETIC_AND_LYMPHOID_TISSUE	0	12.17198
HRT18_LARGE_INTESTINE	0	NA
HS274T_BREAST	0	4.30097
HS294T_SKIN	0	6.08691
HS571T_OVARY	0	NA
HS578T_BREAST	0	4.34381
HS604T_HAEMATOPOIETIC_AND_LYMPHOID_TISSUE	0	NA
HS611T_HAEMATOPOIETIC_AND_LYMPHOID_TISSUE	0	16.4359
HS683_CENTRAL_NERVOUS_SYSTEM	0	9.38945
HS695T_SKIN	0	11.10215
HS706T_BONE	0	5.60788
HS729_SOFT_TISSUE	0	7.85322
HS746T_STOMACH	0	16.79801
HS751T_HAEMATOPOIETIC_AND_LYMPHOID_TISSUE	0	5.75698
HS766T_PANCREAS	0	10.3132
HS852T_SKIN	0	6.22814
HS936T_SKIN	0	7.47664
HS939T_SKIN	0	8.99696
HS944T_SKIN	0	6.40887
HSC2_UPPER_AERODIGESTIVE_TRACT	0	6.11291
HSC3_UPPER_AERODIGESTIVE_TRACT	0	5.49146
HSC4_UPPER_AERODIGESTIVE_TRACT	0	5.84632
HT_HAEMATOPOIETIC_AND_LYMPHOID_TISSUE	0	18.59053
HT1080_SOFT_TISSUE	0	10.70682
HT115_LARGE_INTESTINE	0	14.08056
HT1197_URINARY_TRACT	0	7.82588
HT1376_URINARY_TRACT	0	8.91013
HT144_SKIN	0	9.60628
HT29_LARGE_INTESTINE	0	11.00117
HT55_LARGE_INTESTINE	0	4.59001
HTK_HAEMATOPOIETIC_AND_LYMPHOID_TISSUE	0	NA
HUCCT1_BILIARY_TRACT	0	0.91112
HUG1N_STOMACH	0	9.42901
HUH1_LIVER	0	9.36497
HUH28_BILIARY_TRACT	0	4.23022
HUH6_LIVER	0	8.83529
HUH7_LIVER	0	13.3707
HUNS1_HAEMATOPOIETIC_AND_LYMPHOID_TISSUE	0	17.92074
HUPT3_PANCREAS	0	9.45525
HUPT4_PANCREAS	0	9.33544
HUT102_HAEMATOPOIETIC_AND_LYMPHOID_TISSUE	0	8.91272
HUT78_HAEMATOPOIETIC_AND_LYMPHOID_TISSUE	0	7.28727
HUTU80_SMALL_INTESTINE	0	11.69783
IALM_LUNG	0	4.84743
IGR1_SKIN	0	12.6279
IGR37_SKIN	-2	9.70622
IGR39_SKIN	0	6.7976
IGROV1_OVARY	0	12.74476
IM95_STOMACH	0	16.12916
IMR32_AUTONOMIC_GANGLIA	-2	7.68401
IPC298_SKIN	-2	6.0369

ISHIKAWAHERAKLIO02ER_ENDOMETRIUM	0	10.96282
ISTMES1_PLEURA	0	5.31647
ISTMES2_PLEURA	0	8.36381
J82_URINARY_TRACT	0	11.4377
JEKO1_HAEMATOPOIETIC_AND_LYMPHOID_TISSUE	0	14.59073
JHESOAD1_OESOPHAGUS	0	12.24673
JHH1_LIVER	0	5.16028
JHH2_LIVER	0	3.87166
JHH4_LIVER	0	9.42579
JHH5_LIVER	0	7.69127
JHH6_LIVER	0	6.68073
JHH7_LIVER	0	14.82614
JHOC5_OVARY	2	14.47768
JHOM1_OVARY	0	2.98191
JHOM2B_OVARY	0	9.71751
JHOS2_OVARY	0	4.85322
JHOS4_OVARY	2	14.47184
JHUEM1_ENDOMETRIUM	0	6.51265
JHUEM2_ENDOMETRIUM	0	11.29363
JHUEM3_ENDOMETRIUM	0	5.31767
JHUEM7_ENDOMETRIUM	0	6.19352
JIMT1_BREAST	0	10.41902
JJN3_HAEMATOPOIETIC_AND_LYMPHOID_TISSUE	0	11.72254
JK1_HAEMATOPOIETIC_AND_LYMPHOID_TISSUE	0	5.06072
JL1_PLEURA	0	6.38714
JM1_HAEMATOPOIETIC_AND_LYMPHOID_TISSUE	0	17.45421
JMSU1_URINARY_TRACT	0	11.42219
JURKAT_HAEMATOPOIETIC_AND_LYMPHOID_TISSUE	0	13.94415
JURLMK1_HAEMATOPOIETIC_AND_LYMPHOID_TISSUE	0	16.95113
JVM2_HAEMATOPOIETIC_AND_LYMPHOID_TISSUE	0	4.76026
JVM3_HAEMATOPOIETIC_AND_LYMPHOID_TISSUE	0	7.01206
K029AX_SKIN	0	7.42375
K562_HAEMATOPOIETIC_AND_LYMPHOID_TISSUE	0	13.80869
KALS1_CENTRAL_NERVOUS_SYSTEM	0	9.98689
KARPAS299_HAEMATOPOIETIC_AND_LYMPHOID_TISSUE	0	7.81416
KARPAS422_HAEMATOPOIETIC_AND_LYMPHOID_TISSUE	0	8.79628
KARPAS620_HAEMATOPOIETIC_AND_LYMPHOID_TISSUE	0	18.44067
KASUMI1_HAEMATOPOIETIC_AND_LYMPHOID_TISSUE	0	10.37638
KASUMI2_HAEMATOPOIETIC_AND_LYMPHOID_TISSUE	0	15.22145
KASUMI6_HAEMATOPOIETIC_AND_LYMPHOID_TISSUE	0	9.05755
KATOIII_STOMACH	0	11.51693
KCL22_HAEMATOPOIETIC_AND_LYMPHOID_TISSUE	0	8.35169
KE37_HAEMATOPOIETIC_AND_LYMPHOID_TISSUE	0	11.83361
KE39_STOMACH	0	8.09516
KE97_HAEMATOPOIETIC_AND_LYMPHOID_TISSUE	0	16.15403
KELLY_AUTONOMIC_GANGLIA	0	19.28533
KG1_HAEMATOPOIETIC_AND_LYMPHOID_TISSUE	0	12.7306
KG1C_CENTRAL_NERVOUS_SYSTEM	0	3.01078
KHM1B_HAEMATOPOIETIC_AND_LYMPHOID_TISSUE	0	4.96908
KIJK_HAEMATOPOIETIC_AND_LYMPHOID_TISSUE	-2	7.19644
KLE_ENDOMETRIUM	0	8.06353
KM12_LARGE_INTESTINE	0	12.22116
KMBC2_URINARY_TRACT	0	8.59126
KMH2_HAEMATOPOIETIC_AND_LYMPHOID_TISSUE	0	12.73178
KMM1_HAEMATOPOIETIC_AND_LYMPHOID_TISSUE	0	25.47299
KMRC1_KIDNEY	0	2.14351
KMRC2_KIDNEY	0	3.33386
KMRC20_KIDNEY	0	15.16076
KMRC3_KIDNEY	0	3.74589
KMS11_HAEMATOPOIETIC_AND_LYMPHOID_TISSUE	0	19.92963
KMS12BM_HAEMATOPOIETIC_AND_LYMPHOID_TISSUE	0	9.17627
KMS18_HAEMATOPOIETIC_AND_LYMPHOID_TISSUE	0	9.27592
KMS20_HAEMATOPOIETIC_AND_LYMPHOID_TISSUE	-2	7.07658
KMS21BM_HAEMATOPOIETIC_AND_LYMPHOID_TISSUE	0	6.19919
KMS26_HAEMATOPOIETIC_AND_LYMPHOID_TISSUE	2	16.84564
KMS27_HAEMATOPOIETIC_AND_LYMPHOID_TISSUE	0	10.02203
KMS28BM_HAEMATOPOIETIC_AND_LYMPHOID_TISSUE	0	9.99232
KMS34_HAEMATOPOIETIC_AND_LYMPHOID_TISSUE	0	10.96441
KNS42_CENTRAL_NERVOUS_SYSTEM	0	7.24163
KNS60_CENTRAL_NERVOUS_SYSTEM	0	7.1475
KNS62_LUNG	0	5.43559
KNS81_CENTRAL_NERVOUS_SYSTEM	0	5.35
KO52_HAEMATOPOIETIC_AND_LYMPHOID_TISSUE	0	13.29101
KOPN8_HAEMATOPOIETIC_AND_LYMPHOID_TISSUE	0	13.73186
KP2_PANCREAS	0	6.91769
KP3_PANCREAS	0	9.2873
KP4_PANCREAS	0	8.13689
KPL1_BREAST	0	19.74466
KPNRTBM1_AUTONOMIC_GANGLIA	0	14.33513

KPNSI9S_AUTONOMIC_GANGLIA	0	4.32601
KPNYN_AUTONOMIC_GANGLIA	0	14.28578
KS1_CENTRAL_NERVOUS_SYSTEM	0	7.78842
KU1919_URINARY_TRACT	0	18.08225
KU812_HAEMATOPOIETIC_AND_LYMPHOID_TISSUE	0	6.16018
KURAMOCHI_OVARY	0	3.69214
KYM1_SOFT_TISSUE	0	13.20411
KYO1_HAEMATOPOIETIC_AND_LYMPHOID_TISSUE	-2	4.60453
KYSE140_OESOPHAGUS	0	8.6818
KYSE150_OESOPHAGUS	0	5.85992
KYSE180_OESOPHAGUS	0	14.10933
KYSE270_OESOPHAGUS	0	6.52725
KYSE30_OESOPHAGUS	0	1.41123
KYSE410_OESOPHAGUS	0	9.59829
KYSE450_OESOPHAGUS	0	6.85882
KYSE510_OESOPHAGUS	0	10.35599
KYSE520_OESOPHAGUS	0	3.4652
KYSE70_OESOPHAGUS	0	1.55759
L1236_HAEMATOPOIETIC_AND_LYMPHOID_TISSUE	0	8.79597
L33_PANCREAS	0	8.11896
L363_HAEMATOPOIETIC_AND_LYMPHOID_TISSUE	0	6.84335
L428_HAEMATOPOIETIC_AND_LYMPHOID_TISSUE	0	16.01905
L540_HAEMATOPOIETIC_AND_LYMPHOID_TISSUE	0	11.99903
LAMA84_HAEMATOPOIETIC_AND_LYMPHOID_TISSUE	0	9.96152
LC1F_LUNG	0	5.66362
LC1SQSF_LUNG	0	NA
LCLC103H_LUNG	0	7.61867
LCLC97TM1_LUNG	0	10.03353
LI7_LIVER	0	6.59864
LK2_LUNG	0	31.71921
LMSU_STOMACH	0	12.62729
LN18_CENTRAL_NERVOUS_SYSTEM	0	17.19791
LN229_CENTRAL_NERVOUS_SYSTEM	0	16.61438
LN235_CENTRAL_NERVOUS_SYSTEM	0	8.54417
LN319_CENTRAL_NERVOUS_SYSTEM	0	19.6892
LN340_CENTRAL_NERVOUS_SYSTEM	0	6.12251
LN382_CENTRAL_NERVOUS_SYSTEM	0	8.13866
LN443_CENTRAL_NERVOUS_SYSTEM	0	21.03659
LNCAPCLONEFGC_PROSTATE	0	5.98893
LNZ308_CENTRAL_NERVOUS_SYSTEM	0	9.65179
LOUCY_HAEMATOPOIETIC_AND_LYMPHOID_TISSUE	-2	13.06187
LOUNH91_LUNG	0	9.83673
LOVO_LARGE_INTESTINE	0	17.08131
LOXIMVI_SKIN	0	8.43984
LP1_HAEMATOPOIETIC_AND_LYMPHOID_TISSUE	0	1.09918
LS1034_LARGE_INTESTINE	0	13.61174
LS123_LARGE_INTESTINE	0	4.60597
LS180_LARGE_INTESTINE	0	12.30004
LS411N_LARGE_INTESTINE	0	13.38393
LS513_LARGE_INTESTINE	0	8.55733
LU65_LUNG	-2	4.20248
LU99_LUNG	0	11.74627
LUDLU1_LUNG	0	12.22613
LXF289_LUNG	0	3.2009
M059K_CENTRAL_NERVOUS_SYSTEM	0	14.3869
M07E_HAEMATOPOIETIC_AND_LYMPHOID_TISSUE	0	10.38536
MALME3M_SKIN	0	4.61606
MC116_HAEMATOPOIETIC_AND_LYMPHOID_TISSUE	0	35.54389
MCAS_OVARY	0	8.27271
MCF7_BREAST	0	11.60281
MDAMB134VI_BREAST	-2	4.81447
MDAMB157_BREAST	-2	3.56587
MDAMB175VII_BREAST	0	4.43706
MDAMB231_BREAST	0	10.37275
MDAMB361_BREAST	2	14.31283
MDAMB415_BREAST	0	6.72023
MDAMB435S_SKIN	0	5.04055
MDAMB436_BREAST	0	14.51652
MDAMB453_BREAST	0	8.86122
MDAMB468_BREAST	0	8.73719
MDAPCA2B_PROSTATE	0	8.90286
MDST8_LARGE_INTESTINE	0	9.86361
ME1_HAEMATOPOIETIC_AND_LYMPHOID_TISSUE	-2	4.7784
MEC1_HAEMATOPOIETIC_AND_LYMPHOID_TISSUE	0	12.87577
MEG01_HAEMATOPOIETIC_AND_LYMPHOID_TISSUE	0	9.6536
MELHO_SKIN	0	7.34634
MELJUSO_SKIN	0	10.68738
MESSA_SOFT_TISSUE	0	7.12119
MEWO_SKIN	0	11.49296

MFE280_ENDOMETRIUM	0	12.45929
MFE296_ENDOMETRIUM	0	14.72226
MFE319_ENDOMETRIUM	0	8.46589
MG63_BONE	0	6.56433
MHHCALL2_HAEMATOPOIETIC_AND_LYMPHOID_TISSUE	0	15.19527
MHHCALL3_HAEMATOPOIETIC_AND_LYMPHOID_TISSUE	0	21.09494
MHHCALL4_HAEMATOPOIETIC_AND_LYMPHOID_TISSUE	0	19.54738
MHHES1_BONE	0	10.00863
MHHNB11_AUTONOMIC_GANGLIA	0	9.51493
MIAPACA2_PANCREAS	0	12.23862
MINO_HAEMATOPOIETIC_AND_LYMPHOID_TISSUE	0	15.48331
MJ_HAEMATOPOIETIC_AND_LYMPHOID_TISSUE	0	16.68188
MKN1_STOMACH	0	4.19889
MKN45_STOMACH	0	13.97729
MKN7_STOMACH	0	5.36574
MKN74_STOMACH	-2	1.37198
ML1_THYROID	0	7.58293
MM1S_HAEMATOPOIETIC_AND_LYMPHOID_TISSUE	-2	11.653
MOGGCCM_CENTRAL_NERVOUS_SYSTEM	0	NA
MOGGUVW_CENTRAL_NERVOUS_SYSTEM	0	NA
MOLM13_HAEMATOPOIETIC_AND_LYMPHOID_TISSUE	0	12.79993
MOLM16_HAEMATOPOIETIC_AND_LYMPHOID_TISSUE	0	12.80348
MOLM6_HAEMATOPOIETIC_AND_LYMPHOID_TISSUE	0	6.21167
MOLP2_HAEMATOPOIETIC_AND_LYMPHOID_TISSUE	0	4.6589
MOLP8_HAEMATOPOIETIC_AND_LYMPHOID_TISSUE	0	5.99684
MOLT13_HAEMATOPOIETIC_AND_LYMPHOID_TISSUE	0	15.05927
MOLT16_HAEMATOPOIETIC_AND_LYMPHOID_TISSUE	0	12.55291
MOLT4_HAEMATOPOIETIC_AND_LYMPHOID_TISSUE	0	NA
MONOMAC1_HAEMATOPOIETIC_AND_LYMPHOID_TISSUE	0	14.95835
MONOMAC6_HAEMATOPOIETIC_AND_LYMPHOID_TISSUE	-2	7.25222
MORCPR_LUNG	0	11.05844
MOTN1_HAEMATOPOIETIC_AND_LYMPHOID_TISSUE	-2	NA
MPP89_PLEURA	0	7.75711
MSTO211H_PLEURA	0	4.99989
MUTZ5_HAEMATOPOIETIC_AND_LYMPHOID_TISSUE	0	12.80621
MV411_HAEMATOPOIETIC_AND_LYMPHOID_TISSUE	0	15.7259
NALM1_HAEMATOPOIETIC_AND_LYMPHOID_TISSUE	0	16.81383
NALM19_HAEMATOPOIETIC_AND_LYMPHOID_TISSUE	0	13.16134
NALM6_HAEMATOPOIETIC_AND_LYMPHOID_TISSUE	0	23.99702
NAMALWA_HAEMATOPOIETIC_AND_LYMPHOID_TISSUE	0	13.52948
NB1_AUTONOMIC_GANGLIA	-2	4.80584
NB4_HAEMATOPOIETIC_AND_LYMPHOID_TISSUE	0	18.06654
NCCSTCK140_STOMACH	0	4.16888
NCIH1048_LUNG	-2	6.66971
NCIH1092_LUNG	0	32.4934
NCIH1105_LUNG	0	20.28783
NCIH1155_LUNG	0	29.13918
NCIH1184_LUNG	0	22.40388
NCIH1299_LUNG	0	21.68354
NCIH1339_LUNG	0	18.97814
NCIH1341_LUNG	-2	12.0807
NCIH1355_LUNG	0	13.11967
NCIH1373_LUNG	0	10.12528
NCIH1385_LUNG	0	6.07456
NCIH1395_LUNG	0	4.55502
NCIH1435_LUNG	-2	7.6022
NCIH1436_LUNG	0	25.49797
NCIH1437_LUNG	0	10.39757
NCIH146_LUNG	0	7.15626
NCIH1563_LUNG	0	3.25346
NCIH1568_LUNG	0	21.21004
NCIH1573_LUNG	0	12.90187
NCIH1581_LUNG	0	23.55346
NCIH1618_LUNG	0	21.71272
NCIH1623_LUNG	0	12.73732
NCIH1648_LUNG	0	9.9741
NCIH1650_LUNG	0	10.89557
NCIH1651_LUNG	0	16.85193
NCIH1666_LUNG	0	3.26867
NCIH1693_LUNG	0	6.78925
NCIH1694_LUNG	0	18.0331
NCIH1703_LUNG	0	11.37916
NCIH1734_LUNG	0	9.36172
NCIH1755_LUNG	0	13.68264
NCIH1781_LUNG	0	11.25966
NCIH1792_LUNG	0	13.15643
NCIH1793_LUNG	0	12.26378
NCIH1836_LUNG	0	27.21219
NCIH1838_LUNG	0	9.61555

NCIH1869_LUNG	0	2.66069
NCIH1876_LUNG	0	26.0953
NCIH1915_LUNG	-2	5.85703
NCIH1930_LUNG	0	22.97966
NCIH1944_LUNG	0	18.26599
NCIH196_LUNG	0	10.30046
NCIH1963_LUNG	0	35.50191
NCIH1975_LUNG	0	10.51365
NCIH2009_LUNG	0	8.41013
NCIH2023_LUNG	0	9.25531
NCIH2029_LUNG	0	9.63204
NCIH2030_LUNG	2	19.47093
NCIH2052_PLEURA	0	9.31393
NCIH2066_LUNG	0	13.73184
NCIH2073_LUNG	0	9.65211
NCIH2081_LUNG	0	17.39446
NCIH2085_LUNG	0	20.76613
NCIH2087_LUNG	0	12.08007
NCIH209_LUNG	0	23.48479
NCIH2106_LUNG	0	24.8445
NCIH211_LUNG	0	16.51466
NCIH2110_LUNG	0	11.56689
NCIH2122_LUNG	0	4.86679
NCIH2126_LUNG	0	7.58999
NCIH2141_LUNG	0	NA
NCIH2170_LUNG	0	15.80486
NCIH2171_LUNG	0	22.19488
NCIH2172_LUNG	0	11.9825
NCIH2196_LUNG	-2	13.75588
NCIH2227_LUNG	0	15.11037
NCIH2228_LUNG	0	7.17265
NCIH226_LUNG	0	5.96447
NCIH2286_LUNG	0	11.65524
NCIH2291_LUNG	0	9.13719
NCIH23_LUNG	0	9.88197
NCIH2342_LUNG	0	9.09335
NCIH2347_LUNG	-2	7.91062
NCIH2405_LUNG	0	8.27983
NCIH2444_LUNG	0	3.74579
NCIH2452_PLEURA	0	9.28001
NCIH28_PLEURA	0	9.00251
NCIH292_LUNG	0	15.75922
NCIH322_LUNG	2	32.76769
NCIH3255_LUNG	0	10.83612
NCIH358_LUNG	0	23.44029
NCIH441_LUNG	0	20.42295
NCIH446_LUNG	0	10.11523
NCIH460_LUNG	0	14.26779
NCIH508_LARGE_INTESTINE	0	4.93403
NCIH510_LUNG	0	9.99421
NCIH520_LUNG	0	11.25989
NCIH522_LUNG	0	9.38787
NCIH524_LUNG	0	25.0511
NCIH526_LUNG	0	12.55807
NCIH596_LUNG	0	7.98614
NCIH647_LUNG	0	8.60952
NCIH650_LUNG	0	6.19068
NCIH660_PROSTATE	0	9.9775
NCIH661_LUNG	0	18.36593
NCIH69_LUNG	0	24.12129
NCIH716_LARGE_INTESTINE	0	16.8038
NCIH727_LUNG	0	17.96651
NCIH747_LARGE_INTESTINE	0	9.27497
NCIH810_LUNG	0	8.61943
NCIH82_LUNG	0	21.0122
NCIH838_LUNG	0	8.34363
NCIH841_LUNG	0	12.42884
NCIH854_LUNG	0	4.33978
NCIH889_LUNG	0	21.19012
NCIH929_HAEMATOPOIETIC_AND_LYMPHOID_TISSUE	0	6.81303
NCIN87_STOMACH	0	10.32329
NCO2_HAEMATOPOIETIC_AND_LYMPHOID_TISSUE	0	7.49167
NH6_AUTONOMIC_GANGLIA	0	12.12907
NIHOVCAR3_OVARY	-2	6.11381
NMCG1_CENTRAL_NERVOUS_SYSTEM	0	7.20114
NOMO1_HAEMATOPOIETIC_AND_LYMPHOID_TISSUE	0	11.19574
NUDHL1_HAEMATOPOIETIC_AND_LYMPHOID_TISSUE	0	4.90726
NUDUL1_HAEMATOPOIETIC_AND_LYMPHOID_TISSUE	0	19.68619
NUGC2_STOMACH	0	5.19549

NUGC3_STOMACH	0	7.01458
NUGC4_STOMACH	0	4.29973
OAW28_OVARY	0	14.50142
OAW42_OVARY	0	7.55949
OC314_OVARY	0	8.73696
OC316_OVARY	0	7.63332
OCIAML2_HAEMATOPOIETIC_AND_LYMPHOID_TISSUE	0	17.98929
OCIAML3_HAEMATOPOIETIC_AND_LYMPHOID_TISSUE	0	8.41925
OCIAML5_HAEMATOPOIETIC_AND_LYMPHOID_TISSUE	0	11.88956
OCILY10_HAEMATOPOIETIC_AND_LYMPHOID_TISSUE	0	NA
OCILY19_HAEMATOPOIETIC_AND_LYMPHOID_TISSUE	0	8.83105
OCILY3_HAEMATOPOIETIC_AND_LYMPHOID_TISSUE	0	28.74036
OCIM1_HAEMATOPOIETIC_AND_LYMPHOID_TISSUE	0	4.77489
OCUM1_STOMACH	0	6.28923
OE19_OESOPHAGUS	0	8.3296
OE21_OESOPHAGUS	0	5.46026
OE33_OESOPHAGUS	0	9.60023
OELE_OVARY	0	18.15297
ONCODG1_OVARY	0	5.97402
ONS76_CENTRAL_NERVOUS_SYSTEM	0	9.95711
OPM2_HAEMATOPOIETIC_AND_LYMPHOID_TISSUE	0	5.66063
OSRC2_KIDNEY	0	7.51711
OUMS23_LARGE_INTESTINE	0	3.66807
OUMS27_BONE	0	NA
OV56_OVARY	-2	2.70337
OV7_OVARY	-2	3.73796
OV90_OVARY	0	17.35524
OVCAR4_OVARY	0	3.61089
OVCAR5_OVARY	0	NA
OVCAR8_OVARY	0	13.01751
OVISE_OVARY	0	4.50324
OVK18_OVARY	0	9.57181
OVKATE_OVARY	-2	2.03945
OVMANA_OVARY	-2	2.88387
OVSAHO_OVARY	0	4.73322
OVTOKO_OVARY	0	8.41092
P12ICHIKAWA_HAEMATOPOIETIC_AND_LYMPHOID_TISSUE	0	17.1592
P31FUJ_HAEMATOPOIETIC_AND_LYMPHOID_TISSUE	0	11.90305
P3HR1_HAEMATOPOIETIC_AND_LYMPHOID_TISSUE	0	21.19052
PANC0203_PANCREAS	0	11.57068
PANC0213_PANCREAS	0	10.04141
PANC0327_PANCREAS	0	4.67899
PANC0403_PANCREAS	0	7.5618
PANC0504_PANCREAS	0	11.03029
PANC0813_PANCREAS	0	8.81129
PANC1_PANCREAS	0	6.29998
PANC1005_PANCREAS	0	25.69127
PATU8902_PANCREAS	0	14.41139
PATU8988S_PANCREAS	0	11.71977
PATU8988T_PANCREAS	0	25.42795
PC14_LUNG	0	6.20244
PC3_PROSTATE	0	5.01284
PCM6_HAEMATOPOIETIC_AND_LYMPHOID_TISSUE	0	NA
PECAPJ15_UPPER_AERODIGESTIVE_TRACT	0	11.58071
PECAPJ34CLONEC12_UPPER_AERODIGESTIVE_TRACT	0	4.64717
PECAPJ41CLONED2_UPPER_AERODIGESTIVE_TRACT	0	4.45318
PECAPJ49_UPPER_AERODIGESTIVE_TRACT	0	6.32011
PEER_HAEMATOPOIETIC_AND_LYMPHOID_TISSUE	0	18.60689
PF382_HAEMATOPOIETIC_AND_LYMPHOID_TISSUE	0	32.62988
PFEIFFER_HAEMATOPOIETIC_AND_LYMPHOID_TISSUE	0	25.42155
PK1_PANCREAS	0	10.41368
PK45H_PANCREAS	0	7.45905
PK59_PANCREAS	0	6.1915
PL21_HAEMATOPOIETIC_AND_LYMPHOID_TISSUE	0	15.88947
PLB985_HAEMATOPOIETIC_AND_LYMPHOID_TISSUE	-2	3.92983
PLCPRF5_LIVER	0	7.20342
PRECLH_PROSTATE	0	13.79688
PSN1_PANCREAS	0	4.89812
QGP1_PANCREAS	0	4.8358
RAJI_HAEMATOPOIETIC_AND_LYMPHOID_TISSUE	0	28.07769
RCC10RGB_KIDNEY	0	4.02382
RCHACV_HAEMATOPOIETIC_AND_LYMPHOID_TISSUE	0	19.77257
RCM1_LARGE_INTESTINE	0	7.16126
RD_SOFT_TISSUE	0	9.07205
RDES_BONE	0	15.80603
REC1_HAEMATOPOIETIC_AND_LYMPHOID_TISSUE	-2	5.44043
REH_HAEMATOPOIETIC_AND_LYMPHOID_TISSUE	0	16.13621
RERFGC1B_STOMACH	0	13.35471
RERFLCAD1_LUNG	0	9.51513

RERFLCAD2_LUNG	0	6.00886
RERFLCAI_LUNG	0	9.88447
RERFLCKJ_LUNG	0	5.47035
RERFLCMS_LUNG	0	5.297
RERFLCSQ1_LUNG	2	9.30456
RH18_SOFT_TISSUE	0	1.25535
RH30_SOFT_TISSUE	0	9.08105
RH41_SOFT_TISSUE	0	11.15155
RI1_HAEMATOPOIETIC_AND_LYMPHOID_TISSUE	0	24.4393
RKN_SOFT_TISSUE	0	5.32322
RKO_LARGE_INTESTINE	0	25.02063
RL_HAEMATOPOIETIC_AND_LYMPHOID_TISSUE	0	18.59968
RL952_ENDOMETRIUM	0	15.91296
RMGI_OVARY	0	2.83198
RMUGS_OVARY	0	10.10201
RPMI6666_HAEMATOPOIETIC_AND_LYMPHOID_TISSUE	0	NA
RPMI7951_SKIN	0	4.3254
RPMI8226_HAEMATOPOIETIC_AND_LYMPHOID_TISSUE	0	5.37369
RPMI8402_HAEMATOPOIETIC_AND_LYMPHOID_TISSUE	0	11.23898
RS411_HAEMATOPOIETIC_AND_LYMPHOID_TISSUE	0	8.33957
RT112_URINARY_TRACT	0	12.09586
RT11284_URINARY_TRACT	0	12.29432
RT4_URINARY_TRACT	0	12.27742
RVH421_SKIN	0	7.06977
S117_SOFT_TISSUE	-2	3.34475
SALE_LUNG	0	20.3175
SBC5_LUNG	0	9.4296
SCABER_URINARY_TRACT	0	5.89449
SCC15_UPPER_AERODIGESTIVE_TRACT	0	2.90435
SCC25_UPPER_AERODIGESTIVE_TRACT	0	3.38623
SCC4_UPPER_AERODIGESTIVE_TRACT	0	4.34398
SCC9_UPPER_AERODIGESTIVE_TRACT	0	11.53595
SCLC21H_LUNG	0	13.76169
SEM_HAEMATOPOIETIC_AND_LYMPHOID_TISSUE	0	18.90829
SET2_HAEMATOPOIETIC_AND_LYMPHOID_TISSUE	0	10.00674
SF126_CENTRAL_NERVOUS_SYSTEM	0	7.08779
SF172_CENTRAL_NERVOUS_SYSTEM	0	9.12628
SF268_CENTRAL_NERVOUS_SYSTEM	0	14.63859
SF295_CENTRAL_NERVOUS_SYSTEM	0	14.33238
SF539_CENTRAL_NERVOUS_SYSTEM	0	6.93008
SF767_CENTRAL_NERVOUS_SYSTEM	0	10.26815
SH10TC_STOMACH	0	7.50398
SH4_SKIN	0	4.15317
SHP77_LUNG	0	17.72091
SHSY5Y_AUTONOMIC_GANGLIA	0	NA
SIGM5_HAEMATOPOIETIC_AND_LYMPHOID_TISSUE	0	19.96457
SIMA_AUTONOMIC_GANGLIA	0	7.96409
SJRH30_SOFT_TISSUE	0	NA
SJSA1_BONE	0	8.75114
SKBR3_BREAST	-2	3.66184
SKCO1_LARGE_INTESTINE	-2	5.2069
SKES1_BONE	0	23.87709
SKHEP1_LIVER	0	6.82441
SKLMS1_SOFT_TISSUE	0	5.11445
SKLU1_LUNG	0	6.3882
SKM1_HAEMATOPOIETIC_AND_LYMPHOID_TISSUE	0	9.81576
SKMEL1_SKIN	0	18.50601
SKMEL2_SKIN	0	NA
SKMEL24_SKIN	0	4.60628
SKMEL28_SKIN	0	5.30607
SKMEL3_SKIN	0	10.44359
SKMEL30_SKIN	0	9.12571
SKMEL31_SKIN	0	7.99992
SKMEL5_SKIN	0	7.53772
SKMES1_LUNG	0	5.86756
SKMM2_HAEMATOPOIETIC_AND_LYMPHOID_TISSUE	-2	4.25989
SKNAS_AUTONOMIC_GANGLIA	-2	3.1471
SKNBE2_AUTONOMIC_GANGLIA	0	9.81259
SKNDZ_AUTONOMIC_GANGLIA	0	23.91254
SKNFI_AUTONOMIC_GANGLIA	0	11.674
SKNMC_BONE	-2	12.14231
SKNO1_HAEMATOPOIETIC_AND_LYMPHOID_TISSUE	0	15.12843
SKNSH_AUTONOMIC_GANGLIA	0	9.13925
SKOV3_OVARY	0	9.51316
SKUT1_SOFT_TISSUE	0	16.47656
SLR20_KIDNEY	0	10.3769
SLR21_KIDNEY	0	7.6844
SLR23_KIDNEY	0	9.04333
SLR24_KIDNEY	0	7.83151

SLR25_KIDNEY	0	10.67298
SLR26_KIDNEY	0	7.93012
SNB75_CENTRAL_NERVOUS_SYSTEM	0	5.10868
SNGM_ENDOMETRIUM	0	3.3716
SNU1_STOMACH	0	13.24904
SNU1033_LARGE_INTESTINE	0	9.62075
SNU1040_LARGE_INTESTINE	0	5.8002
SNU1076_UPPER_AERODIGESTIVE_TRACT	0	2.33674
SNU1077_ENDOMETRIUM	0	3.50009
SNU1079_BILIARY_TRACT	0	5.61501
SNU1105_CENTRAL_NERVOUS_SYSTEM	0	6.10158
SNU119_OVARY	0	5.3122
SNU1196_BILIARY_TRACT	0	10.34623
SNU1197_LARGE_INTESTINE	0	2.97736
SNU1214_UPPER_AERODIGESTIVE_TRACT	0	3.69998
SNU1272_KIDNEY	-2	1.40568
SNU16_STOMACH	0	14.05631
SNU175_LARGE_INTESTINE	0	12.85518
SNU182_LIVER	0	3.46798
SNU201_CENTRAL_NERVOUS_SYSTEM	0	3.83763
SNU213_PANCREAS	0	3.98549
SNU216_STOMACH	0	5.00554
SNU245_BILIARY_TRACT	0	13.36088
SNU283_LARGE_INTESTINE	0	11.78163
SNU308_BILIARY_TRACT	0	14.31614
SNU324_PANCREAS	-2	4.61877
SNU349_KIDNEY	0	3.11948
SNU387_LIVER	0	6.61722
SNU398_LIVER	0	25.96187
SNU407_LARGE_INTESTINE	0	14.45093
SNU410_PANCREAS	0	4.92014
SNU423_LIVER	0	9.41153
SNU449_LIVER	0	9.55391
SNU46_UPPER_AERODIGESTIVE_TRACT	0	9.6882
SNU466_CENTRAL_NERVOUS_SYSTEM	0	3.77158
SNU475_LIVER	0	8.9596
SNU478_BILIARY_TRACT	0	12.49376
SNU489_CENTRAL_NERVOUS_SYSTEM	0	1.53068
SNU5_STOMACH	0	9.39477
SNU503_LARGE_INTESTINE	0	8.23936
SNU520_STOMACH	0	12.95423
SNU601_STOMACH	0	11.884
SNU61_LARGE_INTESTINE	0	7.48487
SNU620_STOMACH	0	10.38872
SNU626_CENTRAL_NERVOUS_SYSTEM	0	5.47586
SNU668_STOMACH	0	3.77305
SNU685_ENDOMETRIUM	0	4.27091
SNU719_STOMACH	0	14.07828
SNU738_CENTRAL_NERVOUS_SYSTEM	0	9.52676
SNU761_LIVER	0	5.51242
SNU8_OVARY	0	2.97138
SNU81_LARGE_INTESTINE	0	4.8329
SNU840_OVARY	0	10.02325
SNU869_BILIARY_TRACT	-2	3.33574
SNU878_LIVER	0	3.73638
SNU886_LIVER	0	5.27575
SNU899_UPPER_AERODIGESTIVE_TRACT	0	8.90158
SNUC1_LARGE_INTESTINE	0	6.62661
SNUC2A_LARGE_INTESTINE	0	12.67084
SNUC4_LARGE_INTESTINE	0	12.73166
SNUC5_LARGE_INTESTINE	0	13.13105
SQ1_LUNG	0	8.01942
SR786_HAEMATOPOIETIC_AND_LYMPHOID_TISSUE	0	12.63979
ST486_HAEMATOPOIETIC_AND_LYMPHOID_TISSUE	2	34.70389
SU8686_PANCREAS	0	4.26979
SUDHL1_HAEMATOPOIETIC_AND_LYMPHOID_TISSUE	0	13.20713
SUDHL10_HAEMATOPOIETIC_AND_LYMPHOID_TISSUE	0	15.1751
SUDHL4_HAEMATOPOIETIC_AND_LYMPHOID_TISSUE	0	10.53627
SUDHL5_HAEMATOPOIETIC_AND_LYMPHOID_TISSUE	0	28.60425
SUDHL6_HAEMATOPOIETIC_AND_LYMPHOID_TISSUE	0	31.69157
SUDHL8_HAEMATOPOIETIC_AND_LYMPHOID_TISSUE	0	16.29211
SUIT2_PANCREAS	0	10.18642
SUPB15_HAEMATOPOIETIC_AND_LYMPHOID_TISSUE	0	22.55791
SUPHD1_HAEMATOPOIETIC_AND_LYMPHOID_TISSUE	0	NA
SUPM2_HAEMATOPOIETIC_AND_LYMPHOID_TISSUE	0	5.53477
SUPT1_HAEMATOPOIETIC_AND_LYMPHOID_TISSUE	0	23.65011
SUPT11_HAEMATOPOIETIC_AND_LYMPHOID_TISSUE	0	14.91386
SW1088_CENTRAL_NERVOUS_SYSTEM	0	7.74365
SW1116_LARGE_INTESTINE	0	5.58651

SW1271_LUNG	-2	13.32574
SW1353_BONE	0	5.72321
SW1417_LARGE_INTESTINE	0	9.61489
SW1463_LARGE_INTESTINE	0	6.92087
SW1573_LUNG	0	14.50324
SW1710_URINARY_TRACT	-2	3.36204
SW1783_CENTRAL_NERVOUS_SYSTEM	0	6.15721
SW1990_PANCREAS	0	8.49136
SW403_LARGE_INTESTINE	0	9.79587
SW48_LARGE_INTESTINE	0	13.45929
SW480_LARGE_INTESTINE	0	4.54513
SW579_THYROID	0	8.71522
SW620_LARGE_INTESTINE	0	12.30382
SW780_URINARY_TRACT	0	17.92391
SW837_LARGE_INTESTINE	0	10.13121
SW900_LUNG	0	12.60861
SW948_LARGE_INTESTINE	0	8.50388
T24_URINARY_TRACT	0	17.25765
T3M10_LUNG	0	6.74266
T3M4_PANCREAS	0	11.90331
T47D_BREAST	0	11.51434
T84_LARGE_INTESTINE	0	7.9754
T98G_CENTRAL_NERVOUS_SYSTEM	0	8.3503
TALL1_HAEMATOPOIETIC_AND_LYMPHOID_TISSUE	0	19.29469
TC32_BONE	0	15.06862
TC71_BONE	0	12.86892
TCCPAN2_PANCREAS	0	3.45233
TCCSUP_URINARY_TRACT	0	12.61403
TE1_OESOPHAGUS	0	6.32381
TE10_OESOPHAGUS	0	13.26862
TE11_OESOPHAGUS	0	6.48052
TE14_OESOPHAGUS	0	4.59577
TE15_OESOPHAGUS	0	7.42295
TE4_OESOPHAGUS	0	9.69968
TE441T_SOFT_TISSUE	-2	8.21235
TE5_OESOPHAGUS	0	8.2399
TE6_OESOPHAGUS	0	6.24057
TE617T_SOFT_TISSUE	0	12.65869
TE8_OESOPHAGUS	0	7.46109
TE9_OESOPHAGUS	0	7.13734
TEN_ENDOMETRIUM	0	9.46358
TF1_HAEMATOPOIETIC_AND_LYMPHOID_TISSUE	0	8.23985
TGBC11TKB_STOMACH	0	6.59154
THP1_HAEMATOPOIETIC_AND_LYMPHOID_TISSUE	0	18.08027
TM31_CENTRAL_NERVOUS_SYSTEM	0	6.6923
TOLEDO_HAEMATOPOIETIC_AND_LYMPHOID_TISSUE	0	27.62635
TOV112D_OVARY	0	19.30624
TOV21G_OVARY	0	13.62846
TT_OESOPHAGUS	0	5.32027
TT_THYROID	0	7.9783
TT2609C02_THYROID	0	9.65583
TUHR10TKB_KIDNEY	-2	3.8228
TUHR14TKB_KIDNEY	0	3.15934
TUHR4TKB_KIDNEY	0	2.09932
TYKNU_OVARY	0	13.80211
U118MG_CENTRAL_NERVOUS_SYSTEM	0	13.11809
U138MG_CENTRAL_NERVOUS_SYSTEM	0	NA
U178_CENTRAL_NERVOUS_SYSTEM	0	12.97669
U251MG_CENTRAL_NERVOUS_SYSTEM	0	3.0362
U266B1_HAEMATOPOIETIC_AND_LYMPHOID_TISSUE	0	12.21768
U2OS_BONE	0	22.24844
U343_CENTRAL_NERVOUS_SYSTEM	0	2.84911
U87MG_CENTRAL_NERVOUS_SYSTEM	2	9.35293
U937_HAEMATOPOIETIC_AND_LYMPHOID_TISSUE	0	10.05454
UACC257_SKIN	0	8.94473
UACC62_SKIN	0	8.26165
UACC812_BREAST	0	4.56464
UACC893_BREAST	0	11.87652
UMUC1_URINARY_TRACT	0	4.59204
UMUC3_URINARY_TRACT	0	9.25778
UO31_KIDNEY	0	9.003
UOK101_KIDNEY	0	6.59389
UT7_HAEMATOPOIETIC_AND_LYMPHOID_TISSUE	0	NA
VCAP_PROSTATE	-2	3.47763
VMCUB1_URINARY_TRACT	0	10.13132
VMRCLCD_LUNG	0	NA
VMRCLCP_LUNG	0	NA
VMRCRCW_KIDNEY	0	3.07795
VMRCRCZ_KIDNEY	0	8.86758

WM115_SKIN	0	5.60144
WM1799_SKIN	0	10.00677
WM2664_SKIN	0	13.3388
WM793_SKIN	0	8.40401
WM88_SKIN	0	6.55126
WM983B_SKIN	0	10.7341
WSUDLCL2_HAEMATOPOIETIC_AND_LYMPHOID_TISSUE	0	25.81768
YAPC_PANCREAS	0	8.29151
YD10B_UPPER_AERODIGESTIVE_TRACT	0	6.1273
YD15_SALIVARY_GLAND	0	11.92554
YD38_UPPER_AERODIGESTIVE_TRACT	0	7.51565
YD8_UPPER_AERODIGESTIVE_TRACT	0	4.37154
YH13_CENTRAL_NERVOUS_SYSTEM	0	6.11106
YKG1_CENTRAL_NERVOUS_SYSTEM	0	5.44303
ZR751_BREAST	0	8.8867
ZR7530_BREAST	-2	2.65911

Table S2. Primers used for nuclease assay and Sanger sequencing after FANCA gene editing

Name	Sequence	Application
P_GM_Forward3	TGCTCCTTTTGTGTCATGGGA	Nuclease assay
P_GM_Reverse3	TGCTGGTGTCTTACTCTCTGC	Nuclease assay
P_GM_Forward4	CCTTTGCATCTATTCTCCCCGT	Sanger seq

Table S3. IC50 values for MMC in parental, non-edited, edited, and FANCA-complemented clones from CAL27 and CAL33

Cell line	IC50 MMC	ratio parental/clon
CAL27 (parental)	1.704	
CAL27-c27	1.600	1.065
CAL27-c34	0.129	13.220
CAL27-c34-FANCA	1.602	1.064
CAL27-c47	0.165	10.340
CAL27-c47-FANCA	0.990	1.721
CAL33 (parental)	12.490	
CAL33-c18	12.260	1.019
CAL33-c5	1.298	9.622
CAL33-c5-FANCA	12.370	1.010
CAL33-c11	1.198	10.426
CAL33-c11-FANCA	12.140	1.029

IC50 values are defined as the concentration of drug causing a decrease of 50% of cell viability upon clonogenic assays, cristal violet staining and quantification. Data are means from three different experiments for each cell line.
